# Supplementary material for: Content-rich biological network constructed by mining PubMed abstracts
Source: BMC Bioinformatics. 2004 Oct 8;5:147. doi: 10.1186/1471-2105-5-147 (PMC528731; doi:10.1186/1471-2105-5-147)
Supplement: Additional File 2 — The original results of the above study (non-essential files are deleted to keep the file size under the limit set by BMC bioinformatics). [file 1471-2105-5-147-S2.bz2 › chilibotAdditionalFile2/dip05/746ID7744737E18126/html/TOLA_TOLQ.html]

 


 **TOLA** and **TOLQ** 
  
Found 36 abstracts in PubMed, retrieved 05.  
 

 What does Google say? 
 PDF only 
| .edu only 

---

**Interactive relationship** (e.g. stimulation, inhibition, etc)

**Non-interactive relationship** (e.g. studied together, co-existance, homology, etc.)

- The Tol Pal system of Escherichia coli is composed of the YbgC,  **TolQ** ,  **TolA** , TolR, TolB, Pal and YbgF proteins.  Ref: 11994151 Mol Microbiol, 2002
- Analysis of polar mutations, reverse transcriptase PCR assays, and transcriptional fusion constructs with a promoterless lacZ gene revealed that the genes are arranged in two operons orf1  **tolQ**  tolR  **tolA**  tolB and oprL orf2.  Ref: 12486055 J Bacteriol, 2003
- In Pseudomonas putida, the seven genes, orf1,  **tolQ** , tolR,  **tolA** , tolB, oprL, and orf2, which encode the proteins of this complex, are clustered in a 5.8 kb region of chromosomal DNA.  Ref: 12486055 J Bacteriol, 2003
- Bacteria carrying  **tolA** ,  **tolQ**  and tolR mutations kept their colicin insensitivity as spheroplasts, just as the resistant ones.  Ref: 12094727 Folia Microbiol Praha , 2002
- **TolA** ,  **TolQ** , and TolR are inner membrane proteins, TolB is a periplasmic protein, and Pal, the peptidoglycan associated lipoprotein, is anchored to the outer membrane.  Ref: 11790745 J Bacteriol, 2002
- It was shown that the  **TolA** ,  **TolQ** , and TolR proteins play a critical role in the translocation of group A colicins and filamentous bacteriophage minor coat proteins g3p.  Ref: 11851406 Biochemistry, 2002
